# Integrating gender expertise into the Canadian Armed Forces: challenges for change agents and culture change

**DOI:** 10.3389/fpsyg.2024.1356620

**Published:** 2024-03-20

**Authors:** Victoria Tait-Signal, Angela R. Febbraro

**Affiliations:** Defence Research and Development Canada, Toronto Research Centre, Toronto, ON, Canada

**Keywords:** diversity and inclusion, military organizations, culture change, military culture, prejudice and stigma, gender advisors, gender perspectives, tokenism

## Abstract

**Introduction:**

Gender Advisors (GENADs) have played a key role in the efforts of military organizations worldwide to integrate gender perspectives, and culture change, within the defence and security context. Military organizations, however, continue to face challenges in regard to diversity and inclusion, including limited representation of women and other diverse groups who do not fit the white male, masculine stereotype, and subtle and overt expressions of prejudice and stigma towards under-represented and marginalized groups. In such an organizational context, the integration of gender perspectives has faced challenges, and transformative culture change has remained elusive. In particular, the experience of GENADs suggests that there may be unique challenges to serving as “gender experts” within military organizations. This paper, therefore, examines the lived experience of GENADs within the context of military organizations, as illustrated by GENADs in the Canadian Armed Forces.

**Methods:**

We consider two qualitative studies on the lived experience of GENADs and focus on the shared theme of legitimacy of gender expertise at both individual and systemic levels.

**Results:**

This analysis highlights challenges that gendered power relations may pose for GENADs as individual change agents, and for systemic, transformative culture change, within existing military organizations, while reaffirming the importance of understanding the lived experience of GENADs in their pursuit of more equitable institutional and operational outcomes.

**Conclusion:**

Using social-psychological theories of tokenism, we consider more broadly what it means to be the gender person within masculinized military organizations and conclude with reflections on the potential contours of transformative culture change within the military context.

## 1 Introduction

Gender expertise, unlike expertise in legal or political affairs, is a relatively new construct within military organizations and operations.^1^ Since 2000, Gender Advisors (GENADs) have played a key role in the efforts of military organizations within the United Nations (UN) and the North Atlantic Treaty Organization (NATO) to enhance gender perspectives within military institutions and operations, often referred to as the Women, Peace and Security (WPS) agenda.^2^ UN Security Council Resolution (UNSCR) 1325 is the keystone document in the development of the WPS, and includes four pillars: protection of women and children; prevention of violence against women and children; enhanced participation of women at all levels of the international peace and security architecture; and improved relief and recovery through the adoption of a gender-based lens (see United Nations, 2000). The integration of gender perspectives, thus, involves recognizing that military institutions and operations, including armed conflict, peace operations, and other conflict-affected situations, have different impacts on women, men, and other diverse groups (see Department of National Defence, 2019). Accordingly, the role of military GENADs is to enable militaries to respond to the WPS agenda, to “protect women and girls from the harms of armed conflict, to ensure women’s participation in efforts to build peace and security, and to support gender equality within their own force” (Bastick and Duncanson, 2018, p. 554). As such, the role of GENADs includes the integration of gender perspectives and the protection of gender equality both *externally*, on military operations, and *internally*, within military institutions or organizations (Bastick and Duncanson, 2018; Global Affairs Canada, 2021).

Yet, despite recognition of the importance of gender dynamics, military organizations continue to face challenges in the area of diversity and inclusion, including limited representation of women, members of diverse ethnocultural and racialized groups, and members of other groups who do not fit the white male, masculine stereotype, as well as subtle and overt expressions of prejudice and stigma towards members of under-represented and marginalized groups. Like other military organizations, the Canadian Armed Forces (CAF) is predominately staffed and led by men (Newby and Sebag, 2021). As of July 2023, men comprised approximately 80–86% of CAF Officers and Non-Commissioned Members (NCMs), depending on environment (Army, Navy, Airforce). Women occupied 12 of the 138 positions at General Officer and Flag Officer ranks; these ranks are the highest in the CAF, and Officers at these ranks, in effect, lead the organization (Department of National Defence, 2023a,b).

Since the Chief of Defence Staff (CDS) Directive on UNSCR 1325 was issued in 2016, the Canadian military, like other militaries, has put forward significant effort to ensure that GENADs are available to facilitate the integration, or mainstreaming, of gender perspectives across both the military institution and the operational environment. However, little research exists on the experiences or perspectives of GENADs in the context of this work. Existing research on GENADs in NATO militaries, for instance, suggests that GENADs as “change agents” have achieved some successes internally, in changing military practices and mindsets, in increasing recognition of the relevance of gender in NATO Headquarters, and in initiating conversations about equality and discrimination (Bastick and Duncanson, 2018). Similarly, GENADs have made gains externally, in improving security for women and men in local communities and in increasing women’s participation and empowerment (Bastick and Duncanson, 2018). However, GENADs in NATO militaries have also faced resistance within their own institutions, including lack of command support, as well as inadequate resourcing, preparation, and local contextual knowledge (Bastick and Duncanson, 2018; see also Hurley, 2018; Hardt and von Hlatky, 2020; Holvikivi, 2021; Morrison, 2023).

Accordingly, this paper examines the unique challenges that GENADs may face in their role as “gender experts” and as change agents, in the military institutional and operational context, that is, in raising awareness of the importance of integrating gender perspectives and in protecting gender equality both internally and externally in the defence and security context. ^3^ We explore the lived experience of GENADs with a mind towards the gendered power relations that undergird the acquisition and propagation of gender expertise within a masculinized military context (i.e., a context in which masculinity is equated with dominance and aggression; Hurley, 2018). In so doing, we conceptualize GENAD labour and lived experience as embodied, situated, and contextualized knowledge. We place two research studies on the lived experience of GENADs in conversation, focusing on the shared theme of *legitimacy* of expertise at both the individual psychological level and the systemic organizational level. As we will elaborate, this comparative, qualitative analysis illustrates how legitimacy of expertise is informed by the gendered power relations of military service. Our findings will highlight the centrality of gendered power relations embedded in modern military institutions, reflected in forms of prejudice and stigma, as well as tokenism, associated with GENADs and gender expertise, while reaffirming the importance of learning from the lived experience of security personnel in their pursuit of more equitable institutional and operational outcomes.

We will seek to illuminate some of the challenges that gendered power relations may pose psychologically, for GENADs as individual change agents, and more broadly, for the prospects of systemic, transformative culture change in the military organizational and operational context. Such a transformative change would involve challenging gendered power dynamics both internally within the military institution, and externally within military operations. Ultimately, such a change would involve the use of the military less as a tool for war than as a resource for peace, for the fulsome protection of civilians rendered vulnerable by conflict or disaster, and for the realization of the WPS and broader human security agenda (see Bastick and Duncanson, 2018). However, although the role of GENADs encompasses the integration of gender perspectives both internally within military organizations and externally on military operations, our focus here is to highlight and interrogate gendered power relations primarily within the masculinized military *organization*, and to consider the implications of such power relations specifically for GENADs and their lived experience, as both gender experts and change agents. As we will discuss, the lived experience of GENADs is indeed fraught, as the GENAD role involves both enhancing the gender expertise of military organizations while simultaneously challenging the gendered military status quo through that expertise. In this regard, GENADs are uniquely positioned, as they work for the military institution while concurrently subverting elements of its gendered culture. This may render GENADs more vulnerable to skepticism and derision than their peers who work in areas of expertise such as legal affairs or policy.

Our analysis uses critical feminist theories of lived experience and embodied, situated, contextualized knowledge to explore the production of gender expertise and importantly, of gender experts. This involves a critical review of GENADs as both change agents and as embodied knowers, upon whom the formal and informal rules of military culture and discipline are inscribed. This analysis suggests that, in the instance of GENADs, the knower is not objective, but rather the “knowing subject [is] a historically particular individual who is social, embodied, interested, emotional and rational and whose body, interests, emotions and reason are fundamentally constituted by [their] particular historical context” (Jaggar, 1989, p. 6). As such, the lived experience of individual GENADs suggests that this particular form of labour is shaped by capabilities *and* embodied experience. Further, our analysis considers more broadly what it means to be *the gender person* within a masculinized organization, using theories of tokenism, a form of performativity, as a framework for understanding GENAD lived experience within the masculinized military cultural context (Kanter, 1977; Yoder, 2002; Ferguson, 2015; Henry et al., 2017). Although military organizations may profess to embrace diversity and inclusion initiatives, and may make reformist efforts in their direction, such initiatives may not address underlying power inequities, thus hindering prospects for truly transformative culture change. As such, a gap between rhetoric and reality may exist when it comes to culture change within military organizations (see Bastick and Duncanson, 2018). Given the challenges faced by masculinized organizations that are seeking to integrate gender perspectives and expertise in transformative ways, we conclude with reflections on the potential contours of transformative culture change within the military context.

### 1.1 Background: UNSCR 1325 and gender expertise in the Canadian Armed Forces

Canada played a pivotal role in the creation of the international WPS agenda in 2000. As part of a larger effort in advancing Canada’s human security agenda, Canadian delegates to the United Nations Security Council (UNSC) helped to develop, socialize, and defend UNSCR 1325, the first Security Council resolution concerned with gendered disparities in conflict. Prior to the introduction of UNSCR 1325, a thematic resolution on women had never been considered in the Security Council; if women were mentioned in Security Council resolutions at all, the reference to women was in passing, as “victims” or as a “vulnerable group” (Cohn, 2004, as cited in Shepherd, 2008, p. 391). Therefore, when UNSCR 1325 was introduced, it was not simply a formalized attempt to integrate women into global peace and security architecture; it also claimed to challenge the masculine knowledge that informed military organizations around the world (Otto, 2010). Immediately after the adoption of UNCR 1325, Canada formed the “Friends of 1325,” an initiative to ensure that UNSCR 1325 maintains the strength and expertise required to transform the gender culture of the UN and its member states (Tryggestad, 2009, p. 540). Having championed UNSCR 1325 internationally throughout its tenure on the Security Council (1999–2000), Canada has moved towards domestic implementation of the resolution.

Canada has produced three National Action Plans (NAPs) since the Security Council first requested member states to develop such plans in 2004. Canada’s first NAP (2010–2016) remained limited by the political environment in which it was embedded and proved too unwieldy to monitor effectively (see Tiessen and Carrier, 2015). Canada’s second NAP (2017–2022) provided clearer objectives and acknowledged challenges to the provision of security to women within Canada as well as abroad. Canada’s third NAP (2023–2029), initiated in 2021, included consultations with civil society and relevant experts (Global Affairs Canada, 2021), and is currently being launched. Despite improvements in the quality and content of Canadian NAPs, the advancement of gender expertise within the Department of National Defence (DND) and the CAF remains a challenge. Further, although DND/CAF has acknowledged the need to change its gender culture (particularly amidst the prevalence of sexual misconduct throughout the ranks), transformative culture change within the Canadian defence institution has remained elusive (see, e.g., Arbour, 2022).

Substantive efforts to integrate the WPS agenda into the CAF began in January 2016, with the CDS Directive on UNSCR 1325. In addition to mandating training on “Gender-Based Analysis Plus”^4^ across the DND/CAF, the CDS Directive initiated the creation of GENADs to serve as “specialist advisors for the Commanders responsible for the overall integration of gender perspectives into military planning, execution and evaluation” (CDS Directive, 2016, p. 6). The Canadian military’s implementation of UNSCR 1325 has also been driven by its membership in NATO. Alongside UNSCR 1325, NATO’s Euro-Atlantic Partnership Policy (EAPC) on 1325 in 2007, followed by the first iteration of Bi-Strategic Directive 40–1 in 2009 (revised in 2012, 2017 and 2021), calls for the integration of UNSCR 1325 into NATO’s military command structure (NATO, 2012). The NATO architecture for the implementation of the WPS agenda provides an additional impetus for NATO members to consider gender perspectives, as well as a model for its implementation within military organizations.

Although Canadian military personnel receive training on gender perspectives at various junctures throughout their career, the CAF offers no tailored GENAD training for Canadian military members (see Johnstone and Momani, 2019). Instead, CAF GENADs receive a 2-week course at the Nordic Centre for Gender in Military Operations (NCGM), which is administered by the Swedish Armed Forces. As we elaborate below, several GENADs have suggested that the current suite of gender training available is insufficient for the level of expertise required for their role. Such training limitations are exacerbated by the gendered culture of the military, which further complicates the integration of gender expertise. As has been argued elsewhere, the presence of sexual misconduct can also undermine the legitimacy of the military as a credible force for instituting gender perspectives and expertise (Bastick and Duncanson, 2018; Hurley, 2018; Tait, 2020).

### 1.2 Dynamics of tokenism

In concert with challenges to the advancement of gender expertise highlighted above, the social-psychological dynamics of tokenism also represent a significant issue in masculinized military culture. The sheer numerical dominance of men remains a challenge for those seeking to transform military organizational culture, and is further complicated by the deeply entrenched nature of masculinities and the symbolic dominance of masculinized history and culture within military organizations. Thus, as we explore further below, the dynamics of tokenism are not simply a result of the large numbers of men within military organizations, but are also constituted and embedded within a highly masculinized culture, defined by masculine values such as dominance and aggression (Hurley, 2018). Tokenism, defined by Kanter (1977, p. 965), emerges amidst organizational cultures where group representation is skewed between majority-group “dominants” – those that remain in control of group culture – and minority-group “tokens” – those that become “symbolic representatives of their social category” (see also Childs and Krook, 2008, p. 272). In such contexts, group dynamics may emerge that are damaging to both tokens and to the social cohesion of the organization itself. For example, tokens are highly “visible” in these organizations and, as a result, they may face increased psychological pressure to perform well or to prove themselves (Yoder, 1994, 2002). Further, both token and dominant members may become “polarized,” in which real or perceived group differences are exaggerated. Finally, token members may be subjected to strong socialization pressures to “assimilate” to the existing organizational culture, rather than to transform such culture. This may involve performing in ways that are consistent with existing stereotypes (Yoder, 1994, 2002).

The social-psychological dynamics of tokenism, and their effects, have been examined in a range of military contexts, including the United States Military Academy at West Point (Yoder et al., 1983), the United States Army (Pawelczyk, 2021), and the Portuguese Air Force (Santos et al., 2022). As is the case in other militaries, the gender representation in the CAF would be considered “skewed” in Kanter’s terms, as the ratio of men to women hovers around 85:15, depending on the occupation and element.^5^ GENADs, particularly those who identify as women, are therefore working within an organizational cultural context that may be challenging psychologically and is generally not considered optimal for producing transformative change. In such a context, culture change initiatives that have been established to advance diversity and inclusion within military organizations, or to change gendered culture, including initiatives to facilitate the integration of gender perspectives and expertise, may face particular challenges. This may be especially so if the initiatives do not address tokenism and other power dynamics, or are otherwise mostly performative or symbolic in nature, such as initiatives that have been established mainly to comply with regulations from government or international organizations, or to produce limited culture reform (i.e., to “evolve” the culture), rather to create more fundamental, transformative culture change.

Thus, given the challenges that military organizations may face (e.g., in regard to the under-representation of women and other diverse groups; potential gendered power inequities, prejudice, and stigma; the dynamics of tokenism at individual and systemic levels; the lack of sufficient resourcing and tailored training for the integration of gender perspectives and the GENAD role; and organizational resistance to culture change), this research sought to better understand the unique challenges that GENADs may face as they navigate their role as both “gender experts” and “change agents” in the military institutional and operational context, as well as the implications of these challenges for culture change.^6^

## 2 Method: illuminating the lived experience of military gender experts

For this analysis, we draw from two qualitative interview studies that speak to the lived experience of GENADs in the CAF: Tait (2022) and Thomson and Filardo (2021).^7^ Although the studies share important similarities, there are variations that merit further explanation. Tait (2022) is a doctoral dissertation on the CAF’s interpretation of the WPS agenda, including WPS policy and experiences with gender programming within the military organizational context.^8^ Primary research for this dissertation was conducted using individual, semi-structured interviews, approximately 60 minutes in length. There were two sets of interview questions, one set for civilian and military subject-matter experts (SMEs) on the WPS agenda, and one set for non-expert military personnel. In total, 35 SME participants, and 17 non-expert CAF members, consented to a recorded interview. All 52 interviews were conducted from 2017 to 2019. Recorded interviews were then transcribed by the Principal Investigator, the first author of this paper. Among the 35 SME interviewees, three participants were current or former GENADs, and among the 17 CAF members, two were current or former Gender Focal Points (GFPs). Among the three former GENADs, two identified as women, and one identified as a man; both GFPs identified as men. All were current or former CAF members, and none of the participants disclosed any transgender experience or non-binary gender identity.

Thomson and Filardo (2021) is a contracted Development Research and Development Canada (DRDC) research study, with the second author of this paper as its Scientific Authority. A major purpose of Thomson and Filardo (2021) was to examine the role and function of the GENADs in the CAF and to better understand efforts to integrate gender perspectives into operations. A mixed-methods research study, Thomson and Filardo (2021) used several methods of data collection, both qualitative and quantitative; however, here we focus specifically on the qualitative data collected during recorded, semi-structured interviews conducted with six current or former GENADs and one human terrain analyst, a group that included four individuals who identified as women and three individuals who identified as men; all were current or former CAF members. These interviews were conducted in 2018 and 2019 and were approximately 45 minutes in length. While the DRDC contract report included excerpts from these interviews, the complete set of interview transcripts was made available for subsequent analysis.^9^ A mutual theme of interest between the two studies was the lived experience and perception of legitimacy around the gender expertise of GENADs.^10^

The Tait (2022) and Thomson and Filardo (2021) studies both utilized semi-structured interviews to generate first-person accounts and perspectives regarding the experiences and challenges of implementing the WPS agenda in the Canadian military institutional context, and regarding the role of GENADs in the integration of gender perspectives into operations. The interview data were analyzed using a primarily inductive qualitative, interpretive methodology, informed by principles of grounded theory (see Charmaz, 2006). During this process, interview data were understood in the context of “the historical, social, and situational conditions of [their] production” (Charmaz, 2006, p. 299). In other words, the goal of analysis was not to discover objective truths about the GENAD role or the WPS agenda, but rather to unpack the contextualized patterns or webs of meaning that participants assigned to key gender initiatives, and to strive to understand participants’ experiences in implementing such initiatives, from their own perspective and in their own words. In this analytic approach, participants were treated as *experts of their own experience*, and no efforts were made to correct or to question the veracity of their statements. Qualitative analysis, as such, does not seek to uncover objective reality, or to discover universal, causal, or generalizable principles; rather, qualitative analysis is focused on understanding patterns of meaning reflected in the situated, contextual specificities and particularities of research participants’ experiences, as told from their own perspective (see also Charmaz, 2006).

Although interview questions were used to guide the conversations and to introduce broad research topics (e.g., on gender and military culture), the conversations proceeded organically, and participants were encouraged to elaborate on their experiences when they felt comfortable doing so.^11^ Analysis, thus, involved immersing oneself in (i.e., reading and re-reading) the recorded and transcribed interview material that was generated from these conversations. Transcripts from the two studies were first analyzed separately by question, deductively; analysis then proceeded using inductive social scientific methods to identify key emergent themes reflected in each study, separately. After key themes were identified, transcripts from the two studies were compared to determine commonalities between themes, with an emphasis on the lived experience of GENADs. In comparing the two studies, the frequency of themes was less relevant than the conceptual overlap between the key themes identified in each study. Thus, as we elaborate next, our analysis focused on the conceptual overlap of key themes between the two studies.^12^

## 3 Results: challenges to the legitimacy of GENADs and GENAD expertise

Thomson and Filardo (2021) and Tait (2022) offer two significant repertoires of lived experience in regard to GENADs and gender expertise. A shared theme, reflected in both studies, is the challenge of elevating gender expertise to a position of legitimacy within DND/CAF. GENADs have experienced this challenge at two levels of analysis: the individual psychological level and the broader systemic level of the organization. Here, we describe challenges or resistance to the legitimacy of gender expertise at both levels of analysis. Although gender expertise is a relatively new form of expertise within the CAF, reviews of CAF organizational culture conducted by DND defence scientists over the past two decades have described this culture as reflective of the masculine majority (Febbraro, 2007), as not recognizing the importance of gender (Davis, 2009), and as inhospitable to servicewomen (Davis, 1997). These characterizations of the gendered aspects of CAF culture have been further substantiated by the recent External Review Authority Report (Deschamps, 2015) and the External Independent Comprehensive Review Authority Report (Arbour, 2022).

Notably, the analysis of interview data presented here is not intended to quantify the degree of resistance to gender expertise experienced by GENADs, but rather to illustrate that when resistance emerges, GENADs interviewed for these studies have experienced it primarily through a challenge to their legitimacy, and to the legitimacy of the expertise that they provide on the integration of gender perspectives. As we will elaborate, these challenges may be indicative of the skewed gender culture of military organizations; in broad terms, gender as a salient category of analysis runs contrary to the dominant masculine culture of the military. As such, and as we will illustrate, both the gender expertise itself, and those tasked with disseminating this gender expertise, have experienced resistance to the legitimacy of this expertise; in some cases, this resistance has manifested as suspicion or other forms of denigration by colleagues, and may reflect prejudice and stigma associated with the gender concept or “brand.” Importantly, this resistance may impact GENADs psychologically, at an individual level, but it may also have implications at the broader systemic level, in terms of the prospects or possibilities for culture change within the military organizational context. Indeed, this resistance may be reflective of the token status of gender expertise within masculine military organizations. Accordingly, GENADs, in this analysis, are seen as both potential change agents and tokens within military organizations; and the challenges that GENADs have faced, as gender experts, highlight the deeply gendered nature of legitimacy in the military organizational context. As such, and as we will elaborate, the insights drawn from the interviews contribute to discussions on tokenism within militarized organizational culture, and further our understanding of some of the challenges associated with culture change initiatives that may be more performative or symbolic in nature, rather than truly transformative, within the military organizational context.

Below, we illustrate these challenges through the lived experience of GENADs themselves, as told in their own words. We discuss resistance to gender expertise; the specific gendered challenges of GENAD labour (i.e., around embodiment and the “female voice”); and the issue of tokenism for the “gender person.”

### 3.1 Resistance to gender expertise

The challenges of gender integration and the introduction of gender expertise into NATO forces have been well-explored within the academic literature (Bastick and Duncanson, 2018; Hurley, 2018; Doan and Portillo, 2019; Johnstone and Momani, 2019; Holvikivi, 2021). This body of research points to a central challenge of gender mainstreaming in militaries; new gender norms must “fight their way into institutional thinking, because established goals may compete with the prioritization of gender equality” (Walby, 2005, p. 322). Gender perspectives, therefore, must break through the entrenched, masculinized status quo of military forces. A similar challenge exists in male-dominated civilian organizations, where resistance to the importance of gender may also pervade organizational culture and is reflected in perceptions that organizational leaders lack commitment to the need for change (Lombardo et al., 2009), or that such commitments are merely symbolic or performative. Such themes were evident in both Thomson and Filardo (2021) and Tait (2022). In both studies, GENADs detailed resistance to gender at a conceptual level, among both peers and superiors. In Tait (2022), a GENAD participant shared that,

Military people don’t necessarily care about prevention and protection; they want to hear about pointy military things … I understand GBA Plus, but in my assessment, at the operational level, to have any kind of success or anything that we can kind of influence, it’s not really about the internal organizational stuff. That’s not for me to be able to influence really. (p. 172)

The GENAD quoted above indicated that the concept of gender did not resonate with military personnel, many of whom failed to see the importance of gender within the military’s environment, particularly at the “pointy” end of operations. Similar themes of resistance to the relevance of gender were reflected in Bastick and Duncanson’s (2018) study of GENADs in NATO. Likewise, a GENAD interviewed by Thomson and Filardo (2021) stated that the word “gender”:

…makes military people shut down and not really interested in talking about it just because the word is kind of a charged word if you don’t understand the context...a large majority of the people in the CAF have a misunderstanding about what [gender] is. …[T]hey think it’s fluffy woman stuff: employment equity, sexual misconduct, etc. So as soon as I am the person to say, ‘Hi, I’m X, I’m the gender advisor,’ I see faces like....groans. I’ve had to figure out how to connect with operators [and] to get them to put down their barrier to the word gender perspective and kind of try to make sense of it. The way I chose to do it is really focusing on operational effects and not focusing on women and equal rights, etc. (Participant 5)

The studies by Thomson and Filardo (2021) and Tait (2022) both demonstrate that GENADs have experienced resistance to the content of gender expertise, including prejudicial attitudes that it represents “fluffy woman stuff.” Specifically, the content of such expertise was not understood to be a legitimate priority for military personnel. This point is further underscored by a participant in the Thomson and Filardo (2021) study who stated that, to win over a military audience, they would explain, “[L]ook, my job here is not to talk about Operation Honour,^13^ it’s about producing an operational effect that affects all segments of the population equally” (Participant 7). This GENAD, like the previous participant, suggests that gender expertise was not deemed to be sufficiently important on its own; as such, it was necessary for this GENAD to frame their role in terms of producing *operational effects* on all segments of a population *equally*, rather than in terms of focusing on gender. The GENAD went on to state: “In order to get some traction, I frequently said, ‘Look, it’s more than gender. It’s about marginalized populations, it’s about you know, differing ethnicities.’” Although the attention to marginalization and other identity factors beyond gender is important and laudable, within the context of the masculinized culture of the CAF, the statement may also reflect a more limited understanding of the intersectional dynamics of gender that may be prevalent within the organization. Such an understanding would require attention to the intersectionality between gender and race, class, ability, and so forth. Alternatively, the statement may also represent a response to organizational resistance or lack of commitment to the importance of gender in and of itself. Indeed, talking about gender equality *per se* may be perceived as threatening to military audiences and to the masculine military culture (see Bastick and Duncanson, 2018, p. 569).

In some instances, GENADs perceived that there may be a lack of institutional support for the establishment of the GENAD role at the command level, further suggesting the performative nature of the role or related initiatives. Participant 3 in the Thomson and Filardo (2021, p. 8) study recalled the following words from a Commanding Officer: “Oh, you are the Gender Advisor? So, I’m supposed to drop my trousers and you’ll advise me on what gender I am?” The participant went on to say: “I spent between ten and 20 % of my time on that exercise explaining to people that I was not the Op HONOUR person...” This GENAD’s lived experience illustrates that even at the command level, gendered expertise may not be well understood. In practice, relatively few CAF members have been granted the opportunity to participate in GENAD training. Such training is provided at the Nordic Centre for Gender in Military Operations (NCGM) in Sweden, and although all NATO GENADs are expected to attend this course prior to their posting, relatively few CAF members have been able to attend the courses. As of 2019, only four seats per year were available to Canadian military personnel. The DND and CAF’s Progress Report on Women, Peace, and Security (2019–20) highlights the challenges posed by such limited training, and admits that there remains “misunderstanding over roles and responsibilities of GENAD/GFPs that continues to challenge the advancement of operational integration of gender perspectives [and] challenges persist in terms of amount of GENADs/GFPs trained, due to the reliance of foreign training establishments” (Global Affairs Canada, 2021, online). The Progress Report further stated that, “due to limited number of national seats available at the [NCGM] several of our forces deployed without the complete training package” (Global Affairs Canada, 2021, online). Challenges in attaining sufficient training not only result in a poor institutional understanding of the GENAD role, but our research suggests that insufficient training has had an impact on the confidence of individual GENADs, several of whom saw limited training as a threat to their credibility amongst their peers.^14^

GENADs and GFPs in both Thomson and Filardo (2021) and Tait (2022) shared their struggle to be perceived as credible sources of legitimate expertise given the limited gender training that they have received. A GENAD in the Tait (2022) study recalled their operational experience:

[Y]ou’re sitting in a room with a legal advisor who probably has a post-graduate degree in law, this is their life’s work, their profession, they are licensed, you’re there with a public affairs advisor who again, probably 20 years of doing public affairs in the forces in a military capacity, the policy advisors, generally fairly switched on with a huge mechanism of reach-back and support, and then on the tactical sense you have your engineers advisor, your fires advisor, there are artillery or engineer officers with twenty years of…. And you’ve got your GENAD who’s been on the job for you know, four months. (p. 171)

The GENAD quoted above lamented being called on to provide gender expertise with little or no formal education on the subject. Insufficient training undermined the GENAD’s perception of their legitimacy as an expert, and they struggled to grasp the complexity of intersectional gender dynamics. Another GENAD went on to state:

I still have daily frustrations and feel woefully unprepared in two kinds of realms. Number one... these people have spent their whole careers learning how to advise and what to advise and I get from the commander, ‘what do you think?’ …. I don’t know yet how to apply what we’re learning and what we’re talking about to something that a commander would care about….and then my other reason is that yes, we have our CDS Directive from 2016 that says we will integrate gender perspectives in operations but basically then it’s like, over to you guys to figure it out yourselves (Tait, 2022, p. 172).

As the lived experience of these GENADs demonstrates, it is difficult to be perceived as a legitimate source of gender expertise in the absence of a fulsome training program. This was especially frustrating for the GENAD quoted directly above, who shared their experience with the lack of direction from the strategic level. From a psychological perspective, this experience can be uniquely alienating and isolating for GENADs, who themselves are acutely aware of this shortcoming. Within the civilian sector, one gains gender expertise, much like legal expertise, through academic training. In a 2015 study of professional gender experts^15^ Thompson and Prügl found that over 92% of their 118 participants had graduate degrees, including 27% who had attained a PhD. There is a considerable disconnect between civilian and military standards for gender expertise, which suggests that the current suite of gender programming within many military organizations may not be robust enough to confer sufficient expertise, and may undermine the transformative potential of the GENAD role. In general, a lack of training, resources, prioritization, and other forms of institutional support for an organizational initiative (e.g., the integration of gender expertise into military organizations) can be an indicator that the initiative is more symbolic, token, or performative in nature, rather than a catalyst for transformative change (Henry et al., 2017). As we have seen, this token status may be evident at an organizational or systemic level, but it may also be experienced by GENADs at the psychological or individual level, with potential impacts on their self-confidence and sense of legitimacy as an expert. As we discuss next, this may be especially so for GENADs who identify as women.

### 3.2 Embodied experience and the “female voice”

Our interviews indicated that GENADs who identify as women may experience different forms of resistance from GENADs who identify as men. This speaks to the perceived legitimacy of women’s voices within military institutional or operational contexts, and the ways in which women who provide gender expertise, in particular, may be devalued within the masculinized culture of military organizations. Participant 1 from the Thomson and Filardo (2021) study recalled an exercise with German military personnel, stating:

[I]f it’s a female GENAD that’s talking to them they’ll be like “Okay, yeah, we’ve heard your piece, thank you” and then they won’t do it, but if they hear a man say it, they’ll respect the message more, and they’re more willing to do it. (Participant 1)

The GENAD quoted above, who identified as male, explained that in their experience, women do not have the “perceived authority of the male voice...the voices of women are not really listened to as much as the voices of men, just because it’s not perceived as of the same merit, which is unfortunate.” While further research is necessary to substantiate this inference, this GENAD’s experience indicates that the legitimacy of gender expertise may be compromised amongst military organizations when articulated by women.

Indeed, within the lived experience of servicewomen, serving as a GENAD may represent a challenge distinct from that of their male counterparts. Participant 4 from the Thomson and Filardo (2021) study explained,

Additionally, being a woman in the military, and many of my female friends say the same thing, we’re really not interested in talking about how we’re women in the military or how we’re different or special or anything, we really just want to talk about operations and doing our job, so having to be in this role and figure out this role and now being a voice behind it has been challenging. (Participant 4)

This participant indicates that serving in the GENAD role as a woman – a gender identity that is at odds with the dominant culture of military organizations – may be uniquely burdensome. Similar commentary was offered by GENADs in Tait (2022); for example, a GFP reflected that, although she believed in the importance of gender perspectives, she did not believe that gaining and communicating gender expertise would enhance her credibility or career trajectory within her trade. Such experiences mirror those of civilian women working in humanitarian efforts with military men, whereupon gaining credibility was a persistent challenge for women, and in particular, young women (Febbraro, 2015). A participant quoted in Febbraro (2015, p. 289) reflected,

The biggest challenge I’ve had is to...and I’ve been actually told this by military people, which is interesting, I’d come in quite young, new graduate and I’m a woman and I have no military background, and so getting your thoughts heard and actually acknowledged and action[ed] is [a] very difficult process.... I’ve had to master how to...get my thoughts in from the back door. (NGO worker, p. 289)

This excerpt illustrates the complexity of gender dynamics within the masculinized environment of military organizations. These organizations can present a multifaceted challenge to gender expertise that intersects with a number of identity factors, including gender, age, and military experience. Indeed, our findings illustrate the role of gendered power relations in military institutions and operations and underscore the importance of learning from the lived experiences of GENADs, in order to better understand existing military culture. Importantly, our findings also illustrate the complex challenges and resistances faced by GENADs in their role as agents of knowledge – and as change agents – within the masculinized culture of military organizations. As we have seen, such a role can be complicated and compromised by negative attitudes towards and the potential token status of the GENAD role and of gender expertise within the military context.

### 3.3 Our “gender person”

Within the context of international development, Ferguson (2015) outlined the multifaceted challenges of being an organization’s gender expert or *gender person*. In this context, the *gender person* must avoid being perceived as *too academic*, so as not to overburden or alienate senior management with *too much gender*. Yet, the gender person must simultaneously support gender as a concept and work against the marginalization of gender issues. Indeed, this challenge is similar to the difficult balancing act that GENADs must confront within the daily practices of their role. A participant in Thomson and Filardo (2021) reflected on the need to avoid coming across as a “gender warrior” or crusader for “the gender cause,” or risk that people will stop listening:

If you sort of paint yourself as a gender warrior … people think that you’re only there to change how people act and talk; you might lose access [to the community]. People might stop listening to you. We have this training in the military, and it’s called Bystander Training and if you see any inappropriate behaviour, you’re supposed to call it out. [Gender Focal Points] are going to have an additional level of training where they’re going to be more sensitive to it, but there is that risk of coming across as crusading for the gender cause so… There’s a balance that must be struck there. There’s a value and then there’s balance. (Participant 5)

Our participants frequently discussed the difficulty that they faced in raising gender issues, which appear to be somewhat stigmatized, while ensuring that they (as the “gender person”) do not disturb the established discourse of the organization. In that sense, the ability of a GENAD to effect change may be limited. Reflecting on their experience, Participant 7 in the Thomson and Filardo (2021) study recalled that, although CIMIC (Civil Military Cooperation) and STRATCOM (Strategic Communications) personnel^16^ were generally receptive to gender expertise, and the role that it plays in intelligence and information operations, “everybody else was like, oh Jesus Christ, here comes the political correctness police.” These experiences illustrate that to be seen as a credible source of expertise and competence, GENADs must not be associated too closely with the concept of gender.

Given the social-psychological dynamics of tokenism, resistance to change within military organizations, in which the gender ratio is highly skewed, is unsurprising. Reflecting on her experiences of teaching about gender to a military audience, a participant in Tait (2022) commented that,

When you’re teaching [gender] to a military audience [which is] already skeptical and looking for reasons not to buy into what you’re selling, you risk alienating them if they detect a personal agenda, not just passing on information. (p. 201)

Within this excerpt is the belief that knowledge and expertise must be impersonal and value-neutral to be seen as credible or valuable. However, like all knowledge and expertise, gender expertise is inextricably linked to the society in which it is produced, and it is often imbricated in the personal experience of the expert. Despite this, GENADs must refrain from appearing to have a strong personal or political agenda. As earlier highlighted, GENADs who behave like “gender warriors” or as crusaders for the “gender cause” may be viewed negatively, as too disruptive to the dominant masculinized organizational culture of the military organization, and may be particularly constrained in their ability to effect culture change. This interpretation of our interview findings coincides with Kanter’s (1977) observation that “tokens are left with little choice about accepting the culture of the dominants” (see also Childs and Krook, 2008, p. 727). As such, our findings illustrate some of the challenges that gendered power relations, and tokenism, may pose for GENADs as individual change agents, at a psychological level, and for systemic, transformative culture change, at an organizational or institutional level, while reaffirming the importance of understanding the lived experience of GENADs in the pursuit of more equitable organizational and operational outcomes.

## 4 Discussion

Our findings highlight several challenges associated with the process of acquiring and communicating gender expertise within the context of military organizations, including resistance to gender expertise and the dynamics of tokenism. Further, this process may present unique challenges for GENADs, and for GENADs who identify as women, as their gender identity is at odds with the dominant gender culture of military organizations, both numerically and symbolically. This is not to suggest that GENADs who identify as men do not experience challenges in performing the GENAD role; rather, the challenges that men face to their legitimacy may in part be a reflection of their embodied experiences as men in the role (Hurley, 2014, 2018; see next section below). Such lived experiences highlight important specificities in our understanding of the challenges that GENADs have faced regarding the perceived legitimacy of their gender expertise within the masculinist military context (on masculinity in the military, see also Hinojosa, 2010; Koeszegi et al., 2014; Moore, 2017; Taber, 2018; Davis, 2022). While the specific experiences of the GENADs interviewed in Tait (2022) and Thomson and Filardo (2021) were personal and unique, commonalities emerged around the perceived legitimacy of their gender expertise and their credibility as gender experts. These challenges were evident at the individual, psychological level of GENADs’ lived experience, with impacts on self-confidence as legitimate experts; but the challenges also suggested a larger systemic, institutional, and cultural resistance to the content of gender expertise or gender perspectives, which may undermine the potential for more transformative culture change. These commonalities suggest that efforts at integrating gender perspectives into the military context may be more performative, token, or symbolic, than truly transformative, as is evident at both the individual and systemic level; that the power structures embedded within masculinized military culture, including hierarchy and command structures, remain resistant towards gender diversity; and that this resistance complicates the ability for change agents, such as GENADs, to integrate gender perspectives into military institutions and operations. Further, this resistance may also hinder institutional efforts to create a more inclusive culture within military organizations. Overall, our findings echo previous research with GENADs in NATO (e.g., Bastick and Duncanson, 2018). Below, we consider our findings on gender expertise and GENADs in the context of theories of embodied experience and feminist perspectives on women in masculinized organizational cultures, and suggest potential avenues for future investigation.

### 4.1 Gender expertise and embodied experience

Orna Sasson-Levy’s work has highlighted the bodily discursive practices to which women soldiers must conform to attain legitimacy among peers. In examining the subjective experiences of women soldiers, Sasson-Levy (2003, p. 441) highlights that “women soldiers in masculine roles adopt various discursive and bodily identity practices characteristic of male combat soldiers, which signify both resistance to and compliance with the military gender order.” Sasson-Levy’s research is further supported by studies on the bodily experiences of women Israeli soldiers in combat in the Gaza Strip: Harel-Shalev and Daphna-Tekoah (2016, p. 315) illustrate that in the embodied practice of war, women’s bodies become important sites for the negotiation of patriarchal expectations of discipline and domination.

While much research exists on the bodily experiences and practices of combatants who identify as women, less is known about the embodied experiences of gender experts operating within a military environment. As this form of expertise is relatively new within the military context, much remains to be explored and understood about the interaction between GENADs and the masculinized environment in which they work. For instance, beyond the findings presented here, relatively little is known about the psychological impact, for GENADs, of working within environments in which their expertise is resisted, delegitimized, or otherwise devalued (e.g., as “fluffy women stuff”). Research on tokenism, for instance, suggests that adverse psychological impacts, such as stress and isolation, may be of concern (see Yoder, 1994). The work of GENADs is further complicated by the contentious political narratives inscribed on gendered knowledge. While traditional Cartesian objectivity demands that knowledge remain impersonal and value-neutral to be perceived as legitimate, gender expertise demands consideration of the socio-historical context and individual experience, as well as recognition of the connection between individual, embodied experience and social-historical context. As our findings suggest, this situation presents unique challenges for those serving as military GENADs, and perhaps even more so for those who identify as women.

Notably, although Sasson-Levy (2003) and Harel-Shalev and Daphna-Tekoah (2016) examined the experiences of women, we do not hold *gender* as synonymous with *women,* or assume that genders have essential, fixed natures. Instead, we recognize the diversity of gender, and suggest that gender identities may play a unique role in shaping the lived experience, and thus the knowledge and expertise, of GENADs – as well as perceptions of GENADs and their legitimacy – which may either constrain or enable their ability to share this knowledge and expertise with others. Hurley (2014, 2018) explored these challenges with military personnel doing “gender work” within NATO Headquarters. Hurley (2018) highlighted a male-identifying participant’s frequent reference to “putting on gender glasses” (p. 79), which he suggested allowed him to perceive the distinct challenges faced by women and men in conflict environments. However, this conceptualization of gender expertise, as an analytical lens to be worn or taken off, like a pair of eyeglasses, risks the disembodiment of GENADs’ lived experience. As Hurley (2018) further argues, evidence suggests that gender knowledge and expertise is still perceived as being tethered to gendered bodies (p. 81). Similarly, the participant quoted above noted that he felt uniquely visible as a “guy” discussing gendered perspectives (Hurley, 2018, p. 81). Further, as our own interviews also indicated, Hurley also found challenges of credibility or legitimacy associated with the gendered, embodied experience of GENADs who identify as women, such as *Anna*, quoted below:

[H]aving a man in the team really adds to credibility and most of the people in the military are men and having a man working on that – what is often perceived as simply women’s issues – gives a great deal of credibility (Hurley, 2014, p. 146).

Like *Anna*, the participant *Grace* stated that, “what I’m looking for is a male champion to talk about this, because I’m not going to get anywhere” (Hurley, 2014, p. 101). These responses echo the concerns, expressed by our GENAD research participants, that legitimacy within a militarized environment is at least partly informed by gendered power relations within the organization, which may impact perceptions of GENADs as well as their embodied, lived experience. The responses also suggest that GENADs who identify as men may play an important role as allies in this context (see also Hurley, 2023; Yarnell et al., 2023). Overall, Hurley’s (2014, 2018) findings, along with our own, suggest that gender expertise stands apart from expertise in the legal or engineering field, for instance, in dealing directly and explicitly with embodied experience and subjectivity.

### 4.2 Women in masculinized organizational culture

Feminist expertise, like gender expertise, encounters a variety of challenges when confronted with a new organizational culture. Organizational cultures, like national cultures, are founded on a shared historical mythology and language; they create rules; they regulate actors and their behaviours; and most importantly, they identify and exclude outsiders. In this regard, critical engagement with GENAD labour, knowledge, and expertise within military institutions and operations is a particularly fruitful avenue for future feminist analysis. In conceptualizing spaces for change, for instance, Katzenstein (1999) explores anti-norm feminist behavior within institutional spaces such as “most religious denominations, within prison management, the health sector, universities, [and] armed forces” as *protest*, even though women within these organizations rarely resort to civil disobedience or violence (Katzenstein, 1999, p. 47 see also Chappell, 2006). Katzenstein argues that these activities are not merely “resistance to the power of dominant elites; [they are] proactive, assertive, demand-making political activism” (Katzenstein, 1999, p. 48). Accordingly, change agents, like GENADs, can pursue norm-breaking change within masculinized organizations (e.g., as “institutional entrepreneurs;” Bastick and Duncanson, 2018, p. 573); however, they must negotiate a challenging terrain in their efforts to integrate gender perspectives and to bring about such change.

As our research indicates, GENADs occupy a difficult position, as they must introduce gender expertise and, as such, disrupt gender norms within military organizations, all while ensuring that the goal of gender equality is communicated in a way that is accessible and acceptable to current organizational members, who often have limited gender expertise. As Merry (2006) highlights, “new ideas and practices may be ignored, rejected or folded into pre-existing institutions… or they may be subverted: seized and transformed into something quite different…” (p. 40). Challenging institutional and organizational norms is thus necessarily difficult; institutions and organizations are often resistant towards “new” ideas and are often protective of current social rules and norms (Towns, 2012, p. 185).

Further, as we have discussed, the challenge of bringing about transformative change within organizations, including military organizations, may be exacerbated by the numerical dominance of a singular identity group. Indeed, the numerical dominance of men within military organizations, and the potential for tokenism, presents a unique challenge for those seeking to confront existing gender norms within masculinist military organizational culture. In such a context, organizational efforts to bring about culture change may be performative and symbolic, while individuals who have been assigned the GENAD role may be similarly tokenized. Within this context, GENADs as change agents face a difficult dilemma: Pursuing transformative change of the gender culture of the military organization risks further tokenism and may even compromise the perceived value of gender expertise, particularly amongst dominant group members. Accordingly, GENADs must often attenuate the disruptive content of their gender expertise, thus limiting the potential for transformative change. While this attenuation does not preempt the possibility of gradual, incremental change within military organizations, it tempers the transformative potential of gender expertise, and of the GENAD role (see also Bastick and Duncanson, 2018).

Yet, the dilemma that GENADs face does not diminish the need for transformative change; nor does it reduce the need to envision the potential contours of such change. Transformative change to the gender culture of military organizations, for instance, must entail an understanding of the gendered power relations within such organizations and must take steps to disrupt and eliminate them, that is, to “re-gender” military organizations (Duncanson and Woodward, 2016; Bastick and Duncanson, 2018; Tait, 2020). This will involve the authentic embrace of diversity and inclusion initiatives, including initiatives pertaining to the integration of gender perspectives and the GENAD role, that are not merely established for compliance reasons, or that are largely performative, symbolic, or reformist in nature. In addition to having the strong endorsement of leadership – which is minimally essential for transformative change – such initiatives must also reflect a deep understanding of the dynamics of tokenism and power relations; and they must further be founded on a strong base of institutional support, including sufficient resources for relevant and tailored training and education, professional development, and related initiatives. Such initiatives must also be *prioritized as essential* to effective, and equitable, military institutions and operations. Finally, as our analysis suggests, the resistance, stigma, and prejudice associated with the gender concept must also be addressed and eliminated. Such contours of transformative change will require *long-term* organizational commitment and sustained prioritization; indeed, short-term efforts at culture reform or evolution will be insufficient. As such, the requirements for transformative change to the gender culture of military organizations are significant. However, the impetus for such change will remain, as the integration of gender perspectives and expertise into military institutions and operations, and the provision of security to those in need, will remain a necessity (Duncanson, 2009; Duncanson and Woodward, 2016; Bastick and Duncanson, 2018). This necessity also highlights the importance of continued research into the interface between GENAD lived, embodied experience and military organizational culture; and into culture change efforts that transcend performativity and are truly transformative.

Indeed, further research is needed to better understand, from the perspective of GENADs themselves, the challenges that they may face within gendered military organizations regarding tokenism, prejudice, and stigma, or other potential barriers, and how these obstacles to diversity and inclusion may manifest at both psychological and systemic levels. For example, research is needed to better understand how GENADs of all genders perceive their role within the military context, both institutional and operational, and how GENADs view the priority that is placed on this role within military institutions and operations. Likewise, given that individual lived experience, as situated and embodied, is connected to and must be understood within its broader social, cultural, and historical context, there is a need to explore, from the perspective of GENADs, the potential interconnections and entanglements between psychological and systemic manifestations of gendered power relations and performativity. From an intersectional perspective, the role of identity factors beyond gender must also be examined, as multiple and intersectional identities may also shape the lived experience of GENADs in unique and complex ways (see, e.g., George, 2020). Such future research directions have the potential to provide a more fulsome, situated, and nuanced understanding of the lived experience of GENADs, including the implications of this experience for culture change. Likewise, a deeper and broader understanding of the perspectives and experiences of GENADs, and of culture change, would also be enriched by a more comprehensive set of research methods, including quantitative survey research with diverse groups of GENADs, and mixed-methods research studies, to complement the insights gained from the type of qualitative research on lived experience presented here.

### 4.3 Conclusion

GENADs are key to the integration of gender perspectives within military institutions and operations, and to the transformation of gendered military culture. Yet, GENADs face difficult challenges in their role as “gender experts” and “change agents” within military organizations. Our analysis of two qualitative studies on the lived experience of GENADs illuminated several of these challenges, including resistance to the legitimacy of gender expertise, particularly for GENADs who identify as women, prejudice and stigma associated with the gender concept, and the dynamics of tokenism and power inequities within numerically and symbolically male-dominated military organizations. These challenges play out for GENADs at the individual, psychological level, as reflected in negative impacts on their self-confidence and sense of being a legitimate expert. The challenges also play out at broader, systemic, organizational levels, as reflected, for instance, in the lack of sufficient training and education for GENADs. Interconnected, these challenges at both individual and systemic levels reflect the gendered power relations that exist within military organizations, and the performative nature of current change efforts. Ultimately, these challenges must be addressed in order to realize the full potential of GENADs as change agents, both institutionally and operationally, and to transform gendered culture within military organizations.

## Data availability statement

The data analyzed in this study is subject to the following licenses/restrictions: portions of the data used for this manuscript are subject to restrictions in accordance with the Carleton University Research Ethics Board (CUREB). Other portions are protected under the Canadian Government Security Policy (GSP) at the appropriate designation. If an Access to Information Request is made, the Director of Access to Information and Privacy (DAIP) screens the data in accordance with the Privacy Act in order to ensure that individual identities are not disclosed. Requests to access these datasets should be directed to https://www.canada.ca/en/treasury-board-secretariat/services/access-information-privacy/access-information/request-information.html.

## Ethics statement

The studies involving humans were approved by the Human Research Ethics Committee (HREC) of Defence Research and Development Canada (DRDC) and by the Director General Military Personnel Research and Analysis (DGMPRA) Social Science Research Review Board (SSRRB), in accordance with DAOD 50620 and 50621 (SSRRB approval # 1638/17F). The research was also approved by the Carleton University Research Ethics Board (CUREB). The studies were conducted in accordance with the local legislation and institutional requirements. The participants provided their written informed consent to participate in this study.

## Author contributions

VT-S: Writing – original draft, Writing – review & editing, Conceptualization, Data curation, Formal analysis, Investigation, Methodology, Project administration, Resources, Funding acquisition. AF: Writing – original draft, Writing – review & editing, Conceptualization, Investigation, Project administration, Resources, Supervision.

## Footnotes

^1^The views expressed here are solely those of the authors and do not reflect the views and values of Defence Research and Development Canada, the Department of National Defence, or the Canadian Armed Forces.

^2^Parts of this paper were drawn from Tait (2022), a doctoral dissertation in the Political Science Department at Carleton University (Ottawa), and from internal Defence Research and Development Canada reports (Thomson and Filardo, 2021; Tait-Signal and Febbraro, 2023) that are not publicly available or have limited circulation. Portions of this paper were presented to the American Political Science Association (APSA) Annual Meeting & Exhibition, Montreal, Canada, September 15–18, 2022 (Tait and Febbraro, 2022).

^3^Given the lack of systematic definition in the literature, for the purposes of this paper, we define “gender expertise” as expertise that recognizes that armed conflicts, peace operations, and other conflict-affected situations have different impacts on women, men, girls, boys, and other diverse groups, and that recognizes the gender dynamics in potentially all military activities, both institutional and operational. This definition is derived from concepts articulated in the CDS Directive for integrating UNSCR 1325 and Related Resolutions into CAF Planning and Operations (2016), which refers to the integration of gender perspectives into military operations, policies, and programs, including their planning, execution, and evaluation; and which states that Canada is committed to the integration of gender perspectives across all government departments by assessing the implications for women, men, girls, and boys, of any planned action, including the design of policies associated with peace operations, fragile states, and conflict-affected situations. Notably, the CDS Directive also refers to the integration of gender perspectives into NATO force structure and NATO-led operations, underscoring the internal (institutional) and external (operational) relevance of gender perspectives within the international peace and security architecture.

^4^Gender-Based Analysis Plus, or GBA Plus, is an analytical lens used by the Government of Canada to advance gender equality in Canada; it is considered synonymous with applying “gender perspectives.” The “plus” highlights that GBA Plus goes beyond gender to include examination of a range of other intersecting identity factors (e.g., age, education, language, geography, culture, income, etc.) to define various diverse groups. GBA Plus is used to assess the potential impacts of policies, programs or initiatives on diverse groups of women and men, girls and boys, taking into account gender and other identity factors (CDS Directive, 2016).

^5^For instance, in occupations such as medicine and dentistry, the gender representation in the CAF is close to parity, while in combat-facing occupations, the representation is highly skewed (e.g., the representation of women in the combat arms is approximately 5%) (Global Affairs Canada, Holvikivi, 2021).

^6^Notably, at the time that this research was conducted, the role of CAF GENADs was to “advise on gender in operational planning and doctrine, as well as on inclusion and gender equality when working with other nations” (CDS Directive, 2016, p. 2). During this period in the CAF’s implementation of the WPS agenda, three GENAD positions were created: one in the Canadian Joint Operations Command (CJOC), one in the Strategic Joint Staff (SJS) and one in the Canadian Special Operations Forces Command (CANSOFCOM). As such, GENADs were tasked with working across both institutional and operational boundaries, to advise commanding officers on gender dynamics in all spheres of military planning and operations.

^7^The description of the method used for this analysis is drawn from Tait-Signal and Febbraro (2023); additional methodological detail is contained in Tait (2022) and Thomson and Filardo (2021).

^8^This research was approved by the Director General Military Personnel Research and Analysis (DGMPRA) Social Science Research Review Board (SSRRB), in accordance with DAOD 50620 and 50621 (SSRRB approval # 1638/17F). The research was also approved by the Carleton University Research Ethics Board (CUREB).

^9^Transcripts from Tait’s (2022) dissertation were not made available to DRDC due to ethics protocol constraints, but the dissertation was made available to DRDC. The quotations used in this article were drawn from the dissertation, which is available on request from the first author.

^10^Between the two studies, a total of 11 GENADs/GFPs were included among the 59 participants (5 in Tait, 2022, and 6 in Thomson and Filardo, 2021). However, other participants shared their views on the GENAD role, on the legitimacy of gender expertise, or on the CAF’s interpretation of the WPS agenda, and thus provided valuable insight on these topics.

^11^Tait’s (2022) interview questions focused on WPS policy and on experiences with gender programming (e.g., “Do you think having special gender advisory staff is needed and of value to the organization?”) (p. 258). Thomson and Filardo (2021) explored the GENAD/GFP role more specifically (e.g., “Tell me about your own personal experiences reporting and conducting gender-related analysis and assessment. What does that look like? How has it been received? How is it integrated into operational planning? What are some of the challenges you faced? What more do you need to support your role?”) (p. 62).

^12^Given the limitations on the sharing of interview data, the analysis was conducted primarily by the first author of this paper.

^13^Operation HONOUR refers to the CAF effort from 2016–2022 to eliminate sexual misconduct within the military; it is seen as an internal or institutional program (see CDS, 2015).

^14^The absence of more robust gender training is notable given the well-developed suite of other graduate-level training programs within DND/CAF, available through the Canadian Defence Academy.

^15^Thompson and Prügl (2015, p. 9) defined professional gender experts as those persons hired for “gender related work in inter-governmental and international non-governmental organizations.”

^16^In the CAF, CIMIC refers to Civil-Military Cooperation; STRATCOM refers to Strategic Communications.
